# Recruitment and feasibility tool

**DOI:** 10.1186/2043-9113-5-S1-S10

**Published:** 2015-05-22

**Authors:** Jasper van Leeuwen, Anca Bucur, Jeroen Keijser, Brecht Claerhout, Kristof de Schepper, David Perez-Rey, Raul Alonso-Calvo

**Affiliations:** 1 Philips Research Europe, 5656 AE Eindhoven, the Netherlands; 2 Custodix NV, 9830 Sint-Martens-Latem, Belgium; 3 Universidad Politécnica de Madrid, 28040 Madrid, Spain

## Characterisation

Tool, patient recruitment, clinical trial feasibility.

## Tool description

The recruitment and feasibility tool (earlier named Yakobo) was developed in the EURECA project to assess protocol feasibility and to find eligible patients for clinical trials. Protocol feasibility functions analyze the feasibility of a trial protocol by assessing the expected patient enrollment rate of a selection of sites given the protocol’s eligibility criteria, based on “historical” data (existing patient data). It allows for answering questions such as whether inclusion and exclusion criteria are useful for defining the proper study population, whether it is likely that the necessary volume of patients can be recruited in time to collect data with sufficient statistical power and/or the expected duration of a trial.

**Figure 1:**
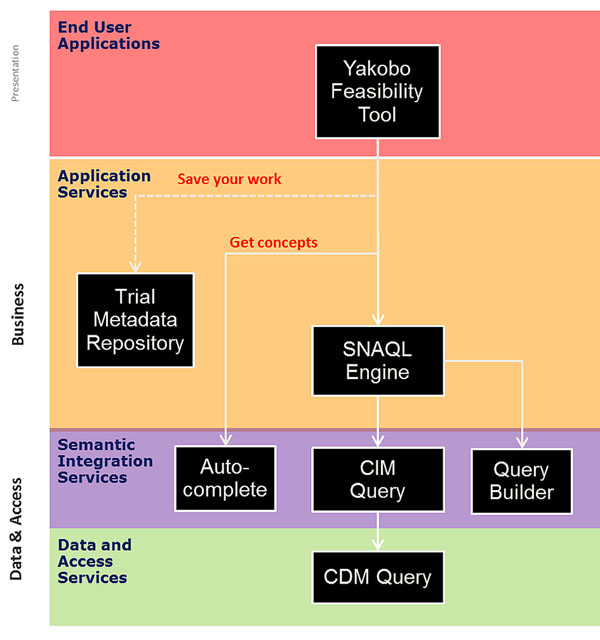
Schema of the workflow for using the Recruitment and feasibility tool.

Criteria are expressed in a domain specific language [1] A Trial metadata repository contains protocol definitions (Figure 1) and the SNAQL engine executes the DSL of the criteria to find patients belonging to the cohort. The SNAQL engine accesses the semantic integration services which provide a query interface [2] with reasoning abilities. Once a clinical protocol has been finalized, the tool is used to find patients eligible for enrollment.

## Status of development

Concept validation and validation by selected user groups.

## Link

http://eurecaproject.eu/
